# Seed shape and size of *Silene latifolia*, differences between sexes, and influence of the parental genome in hybrids with *Silene dioica*

**DOI:** 10.3389/fpls.2024.1297676

**Published:** 2024-03-11

**Authors:** Marcel Hubinský, José Javier Martín-Gómez, Emilio Cervantes, Roman Hobza, Jose Luis Rodríguez Lorenzo

**Affiliations:** ^1^ Department of Plant Developmental Genetics, Institute of Biophysics of the Czech Academy of Sciences, Brno, Czechia; ^2^ National Centre for Biomolecular Research (NCBR), Faculty of Science, Masaryk University, Brno, Czechia; ^3^ Instituto de Recursos Naturales y Agrobiología de Salamanca (IRNASA)-CSIC, Salamanca, Spain

**Keywords:** *Silene latifolia*, *Silene dioica*, seed shape, Morphometrics geometrics, polyploidy, plant hybrid, elliptical Fourier analysis, symmetry

## Abstract

**Introduction:**

Plants undergo various natural changes that dramatically modify their genomes. One is polyploidization and the second is hybridization. Both are regarded as key factors in plant evolution and result in phenotypic differences in different plant organs. In *Silene*, we can find both examples in nature, and this genus has a seed shape diversity that has long been recognized as a valuable source of information for infrageneric classification.

**Methods:**

Morphometric analysis is a statistical study of shape and size and their covariations with other variables. Traditionally, seed shape description was limited to an approximate comparison with geometric figures (rounded, globular, reniform, or heart-shaped). Seed shape quantification has been based on direct measurements, such as area, perimeter, length, and width, narrowing statistical analysis. We used seed images and processed them to obtain silhouettes. We performed geometric morphometric analyses, such as similarity to geometric models and elliptic Fourier analysis, to study the hybrid offspring of *S. latifolia* and *S. dioica*.

**Results:**

We generated synthetic tetraploids of *Silene latifolia* and performed controlled crosses between diploid *S. latifolia* and *Silene dioica* to analyze seed morphology. After imaging capture and post-processing, statistical analysis revealed differences in seed size, but not in shape, between *S. latifolia* diploids and tetraploids, as well as some differences in shape among the parentals and hybrids. A detailed inspection using fluorescence microscopy allowed for the identification of shape differences in the cells of the seed coat. In the case of hybrids, differences were found in circularity and solidity. Overal seed shape is maternally regulated for both species, whereas cell shape cannot be associated with any of the sexes.

**Discussion:**

Our results provide additional tools useful for the combination of morphology with genetics, ecology or taxonomy. Seed shape is a robust indicator that can be used as a complementary tool for the genetic and phylogenetic analyses of *Silene* hybrid populations.

## Introduction

With more than 800 species described, *Silene* is the largest genus in *Caryophyllaceae* (Jafari et al., 2020). *Silene* seeds are characterized by a peripheral position of the embryo (Martin, 1946), anatropous to campylotropous ovules (Johri et al., 2013), and an interesting diversity in seed shape (Yildiz and Cirpici, 1998; Martín-Gómez et al., 2022a; Martín-Gómez et al., 2022b). *Silene* species present a wide adaptation range to environmental conditions, as well as diverse sexual systems and life cycles, including annual and perennial species, and have been considered as a model for ecology and evolution (Bernasconi et al., 2009). Aspects of particular interest include the diversity of reproductive and breeding systems, and sex chromosome evolution (Janoušek et al., 2013). In *Silene*, we found several species used as models for evolutionary studies of sex chromosomes and sex-determining mechanisms (Slancarova et al., 2013). The chromosome number in almost all species of the genus *Silene* is 2x = 24, and there are diverse sexual systems (Casimiro-Soriguer et al., 2015; Balounova et al., 2019). At least three independent origins of genetic sex determination (dioecy and/or subdioecy), as well as a switch from XY to ZW or vice versa, in section *Otites* have been indicated in the last 10 million years during the evolution of dioecy in *Caryophyllaceae* (Slancarova et al., 2013). The genome size of *Silene* species is highly diversified because of numerous DNA amplifications and translocations (Široký et al., 2001), and reports of DNA content are available for a number of *Silene* species (POWO, 2022). Polyploidy in *Silene* can be found in nature (Blackburn, 1933; Popp and Oxelman, 2007; Sheidai et al., 2011). In the case of *Silene latifolia* and *Silene dioica*, polyploidy is absent in nature. On the other hand, synthetic polyploids and haploid plants have been described for both species, and their use has been limited to studying the mechanisms of sex determination and genetic structure of sex chromosomes in different studies (Warmke and Blakeslee, 1939; Westergaard, 1946; Ye et al., 1990; Siroký et al., 1999; He and Horandl, 2022).

Hybridization and polyploidization contribute to plant species diversification. *S. latifolia* and *S. dioica* are two closely related species known for their dimorphic sex determination systems, with males and females having different flower types (Hobza and Widmer, 2008). Both species are native to Europe and the NW. Their hybridization occurs all across the continent and can be found as an invasive species in North America (Blair and Wolfe, 2004; Vercken et al., 2010; POWO, 2022). The presence of natural hybrid populations between *S. latifolia* and *S. dioica* can vary depending on local ecological conditions (Goulson, 2009; Karrenberg et al., 2019). Hybrids between these two species can exhibit intermediate flower colors varying from pure white (typical of *S. latifolia*) to various shades of pink and red (typical of *S. dioica*). The flower color gradient is a well-studied phenomenon for hybrid identification, and together with other flower features and molecular markers, was not sensitive enough to analyze the hybrid zones in detail (Minder et al., 2007).

The shape of plant organs is an important feature of their ecology, and in crops, it is directly related to the yield and quality. Traditionally, seed shape description in *Silene* has been limited to a qualitative comparison with geometric figures based on adjectives (rounded, globular, reniform, or heart shape). Different characteristics of the seed coat cells for several *Silene* species analyzed by scanning electron microscopy (SEM), such as shape or lobe morphology, also have their own set of terms. For the cell suture we have: digitate-long, serrate-short, rectangular and sinuous: for cell lobes there are: smooth, rugose, echinate and papillose (Dadandi and Yildiz, 2015; Hoseini et al., 2017; Martín-Gómez et al., 2022b). Recent work has been published on the seed shape of many species of the genus *Silene*. The seed shape in *Silene* has particular features that can be quantified and compared (Martín-Gómez et al., 2020, 2023). Shape is commonly described as a property of an object invariant under scaling, rotation, or translation. Seed shape quantification has been based on direct measurements such as area, perimeter, length, or width, known as General Morphological Descriptors (GMDs), narrowing statistical analysis. A geometrical approach involves mathematical equations, which encompass morphological traits under complex genetic control. Looking at seed images or their silhouettes, geometric objects are a direct way to achieve mathematical accuracy in seed shape description (Gutiérrez Del Pozo et al., 2020). Geometric Morphometrics (GM) is another quantitative framework for shapes. Different types of sofware are available to automate the protocol from the original JPG black and white binary image to plotted results (Klingenberg, 2008; Bonhomme et al., 2014; Matzig, 2021). Different options include elliptic Fourier analysis (EFA). Using EFA, two-dimensional closed outlines are represented by continuous trigonometric functions, preserving all the shape information in the form of elliptical Fourier descriptors (EFD) (Kuhl and Giardina, 1982; McLellan and Endler, 1998; Cervantes et al., 2022).

An important aspect of shape is symmetry. According to Breno et al. (2013) all life forms are more or less symmetrical; therefore, the study of asymmetry has been used to quantify those stimuli that affect normal development. Bilateral symmetry, in simple terms, is the property of being symmetrical about a vertical plane, and there are three types of asymmetry. Directional asymmetry is a systematic difference between the left and right sides of structures with bilateral symmetry, or systematic differentiation among repeated parts for complex symmetry. Antisymmetry refers to the condition where right- and left-sided forms are equally common within a species, and it is caused by switches between two different states of a trait. Unlike other asymmetries, fluctuating asymmetry denotes small differences between the right and left sides. In simple terms, is the difference between the individual asymmetry and average directional asymmetry (Graham et al., 2010; Benítez et al., 2020). Different quantitative indices achieved through morphological analysis are required for the combination of morphology with genetics, ecology, or taxonomy.

In *S. latifolia*, there is evidence of sexual dimorphism in the vegetative state (Zluvova et al., 2010). Sexual dimorphism in this species is largely restricted to reproductive characteristics, particularly flower number and size, and a positive correlation between flower size, capsule size and seed size has been reported in females (Baker, 1950; Minder et al., 2007). In this study, we analyzed and described the following: 1) differences in size and shape between *S. latifolia* diploid and tetraploid seeds; 2) differences in shape between *S. latifolia* male and female seeds; and 3) differences in seed shape between the hybrids of crosses *S. latifolia* × *S. dioica*, with parental males and females of each species. Finally, differences in the shape of the cells in the dorsal view of seeds in the above genotypes were also tested.

## Materials and methods

### Seed populations

The materials used in this study were obtained from the collection of the Institute of Biophysics in Brno, Czech Republic. An inbred population (U16; 16 generations of full-sib mating) of male and female *S. latifolia* and a population of *S. dioica* collected in Piesky (48° 49’ 3’’ North, 19° 7’ 51’’ East) were used for the tetraploid and hybrid generations in this study. Tetraploid seed generation was performed on female flowers two days after pollination. The whole flower was immersed in colchicine 0.5% plus DMSO for 8 h, followed by several rinses with ddH_2_O. F2 seed offspring were used for imaging analysis. Hybrid seeds were collected after controlled cross-pollination between the male and female plants of both species. Two different crosses, *S. latifolia* female × *S. dioica* male, and four different crosses, *S. latifolia* male × *S. dioica* female genotypes, were used in the controlled crosses. The plants were grown in a greenhouse under standard long-day conditions (22°C, 16 h light/8 h dark). A summary of the plant material and different genotypes used for each multivariate analysis is presented in **Table 1**.

**Table 1:** Summary table of material used in this study.

Species	Multivariate analysis	Seed origin	Items observed	
			Seeds	Cells
***S. dioica***	**Figures 2B–D**, **3A**	Piesky population	20	40
***S. latifolia* 2× (♀ + ♂)**	**Figures 1A**, **2B–D**, **3A**	Inbred population (U16; 16 generations of full-sib mating). IBP collection Acad Sci CzR, Brno.	100	40
***S. latifolia* 2×♀**	**Figure 1A**	Inbred population (U16; 16 generations of full-sib mating). IBP collection Acad Sci CzR, Brno.	34	–
***S. latifolia* 2×♂**	**Figure 1A**	Inbred population (U16; 16 generations of full-sib mating). IBP collection Acad Sci CzR, Brno.	31	–
***S. latifolia* 4× (~♂)**	**Figures 1A**, **2B–D**, **3A**	Three different female and male plants Colchine treated U16 population	80	40
**Hybrids**				
***S. dioica* ♀ × S. *latifolia ♂***	**Figures 2B–D**, **3A**	3 different genotypes 4 independent crosses	Cross 1: 18 Cross 2: 20 Cross 3: 20 Cross 4: 20	40 40 40 40
**S. latifolia ♀ × S. dioica ♂**	**Figures 2B–D**, **3A**	2 different genotypes 2 independent crosses	Cross 1: 16 Cross 2: 16	40 40

For *S. latifolia*, both 2× (♀ + ♂) and 4× (~♂), and for *S. dioica* seed populations, a minimum of four different parental plants were used. For the hybrids all those seeds without defects in the seed coat from the progeny in each individual fertilization event were used. The cell outlines for multivariate analysis were extracted from 10 different seeds. The code for the Multivariate analysis column indicates the exact PCA where the different seed populations were analyzed.

### *S. latifolia* seed sex determination

Under the stereoscope, we started with 100 independent seeds from *S. latifolia* 2× (♀ + ♂) placed in the lateral and dorsal views for previous image acquisition. Immediately after, the seeds were placed, following the same order than in the images, on a filter paper in a Petri dish with 2% agar (plant agar P1001, Duchefa Biochemie). After one week, 65 seedlings from the germinated seeds were collected and frozen in liquid nitrogen. DNA was isolated according to DNeasy (Qiagen, Hilden, Germany), and PCR with sex-specific primers was performed for seed sex determination in *S. latifolia*. Sex-specific primers were used, as described by Hobza et al. (2006). The PCR program was as follows: 95°C for 3 min, 95°C for 5 s, 60°C for 30 s (×40), and a melting step from 72°C to 95°C. This was carried out in a RotorGene (Qiagen, Hilden, Germany) with the qPCR mix SensiFAST™ HRM Kit (Bioline, Meridian Bioscience). DNA from male and female *S. latifolia* was used as a reference. To test for *S. latifolia* 4× (~♂) sex bias, 50 seeds from this genotype, different from those used in the morphometric analysis, were included in this study. The *S. latifolia* 4× plants analyzed here produced predominantly male offspring, as shown in a previous independent test for *S. latifolia* 4× sex bias (unpublished), where 49 embryos were males out of 50 seeds successfully sexed in total. The total number of sexed seeds was 65 (31 males and 34 females), and there were 50 tetraploids (49 males and one female).

### Image acquisition

Lateral and dorsal seed photographs were taken using a stereomicroscope Olympus SZX16 with an objective SDF PLAPO ×0.8, connected to a workstation Quick photoCamera 3.2 (Promicra, CZ). Cell images were obtained using an Olympus Provis AX70 with a ×10 objective under green light (λ = 554 nm) connected to a workstation Isis FISH imaging system (MetaSystems, Germany). Cell images were obtained from the dorsal view of the seeds. This was because the cell shape was more homogenous. Images ranging from 10 to 20 with different foci were taken from the same seed to obtain approximately four to five completely focused cells (depending on the curvature of the area). This process involved cell images from approximately 10 different seeds.

### Image processing

Images were processed using the ImageJ software (Schindelin et al., 2012). The seed pictures were segmented, and outlines from the black and white binary images were used for morphometric analysis. The cell images were stacked using the Extended Depth Field pluin. A focused image was used for cell shape extraction. Segmentation was achieved using WEKA (Arganda-Carreras et al., 2017), and the outline generated with this tool was skeletonized for standardization. The output silhouette image was used for morphometric analysis.

### Morphometric geometrics

General morphological measurements for the individual outlines, including area, perimeter, length, width, circularity, aspect ratio, roundness, and solidity, were calculated by automatic image analysis using ImageJ software (Schindelin et al., 2012). The *J* index is the percentage of similarity between a given seed image and a geometric model and is the result of the ratio: $\;J\;index=\frac{shared\;area}{total\;area}\;$ . (**Supplementary Figure 1**).

The *J* index was calculated by comparing the seed silhouette with lateral models LM1 and LM2 derived from a cardioid, and dorsal models DM2 and DM3 derived from a modified ellipse, according to Martín-Gómez et al. (2020); Juan et al. (2021), and Rodríguez-Lorenzo et al. (2022). For the elliptic Fourier analysis, we started from a binary image with silhouettes, and using the package OutlineR, the outlines were extracted and loaded into the R environment according to Matzig (2021).

**Figure 1:**
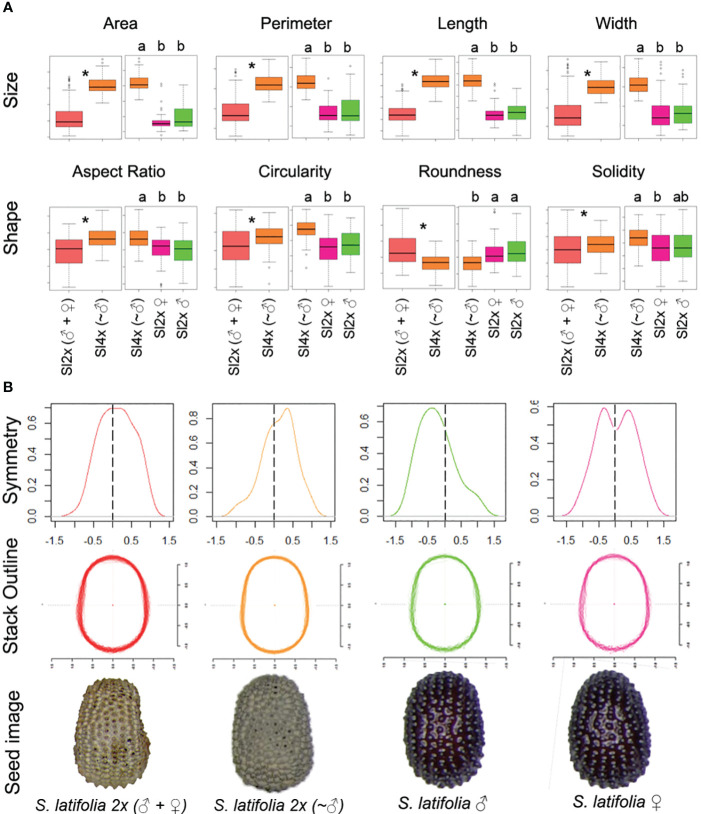
**(A)** Boxplot representation for *S. latifolia* 2x (♀+♂) Vs *S. latifolia* 4x (~♂) (left) and *S. latifolia* 4× (~♂) vs Sex (right) for each general morphological descriptor on the dorsal view of *S. latifolia* seeds. An asterisk indicates statistical differences for *S. latifolia* 2× (♀+♂) vs *S. latifolia* 4× (~♂), and different letters in each individual plot mean statistical differences according to ANOVA and Tukey tests, p-value<0.05. **(B)** Density plot with the symmetry index distribution (up). X axis represents normalized (−1.5, 1.5) index, Y index represents individuals. Scaled and centered outline stack for the dorsal view of *S. latifolia* seeds (middle). X and Y axes represent scaled and centered outline dimensions. Representative example seed image from the dorsal view for each of the genotypes (bottom). Centroid color code: Red, mixed population *S. latifolia* 2× (♀ + ♂); orange, *S. latifolia* 4× (~♂); green, male *S. latifolia*; pink, female *S. latifolia*.

**Figure 2:**
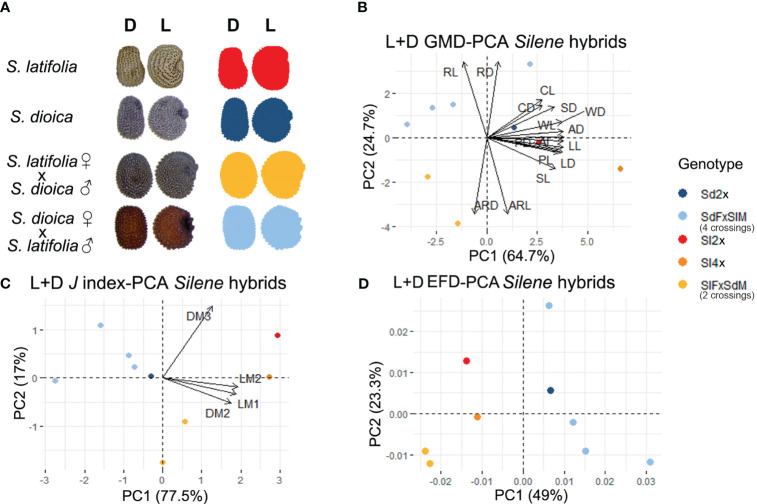
**(A)** Representative example seed image and silhouette from the lateral and dorsal view for hybrids and parents. Principal component analysis from lateral plus dorsal view for: **(B)** general morphological descriptors **(C)** *J* index adjusted to a cardioid or modified ellipse geometric model. **(D)** elliptic Fourier descriptors. Centroid color code: Red-orange applies to *S. latifolia* and hybrids where *S. latifolia* is the maternal genome. Dark-light blue applies to *S. dioica* and hybrids where *S. dioica* is the maternal genome. Name code: Sl, *S. latifolia*; Sd, *S. dioica*; F, female; M, male.

The outlines were processed using the Momocs package and elliptic Fourier descriptors were extracted according to Bonhomme et al. (2014). The elliptic Fourier descriptors used in the statistical analysis corresponded to the number of harmonics that explained >99% of the seed shape. For *S. latifolia* symmetry analysis shown in **Figure 1B**, 42 harmonics were used. For the multivariate analysis of the hybrid seeds shown in **Figure 2**, 44 harmonics were used. Symmetry coefficients were calculated from the descriptors as the ratio of the columns AD (sum of columns A and D) over amp (sum of the absolute values of all harmonic coefficients), according to Iwata et al. (1998). The coefficients for each genotype were normalized (from −1.5 to 1.5) for continuous representation in a density plot. The cell outlines were stacked, centered and scaled with no homologous landmark. Despite differences in shape, the stacked outlines showed high homogeneity (**Supplementary Figure 2**).

### Statistical analysis

Univariate analysis for general morphological measurements was performed using IBM SPSS Statistics v28 (Statistics, 2021). Multivariate analysis was performed using R (R Development Core Team, 2023) for the data from general morphological descriptors and elliptic Fourier descriptors corresponding to seed and seed coat cell outlines. Data met normality and homogeneity of variance assumptions according to the Shapiro–Wilk and Levene tests. Multivariate analysis of variance (MANOVA) was carried out to identify statistical differences in the genotypes used in this study based on the GMD and EFD datasets to support the differences displayed by PCA. An ANOVA followed by a Tukey test was used to identify statistically significant mean differences among the genotypes. Two independent analyses (**Figure 1A**) were performed in comparison with *S. latifolia* 4× (~♂) to avoid interactions between *S. latifolia* 2× (♀ + ♂) and the individual sexes because both sexes are subpopulations of *S. latifolia* 2× (♀ + ♂). Principal component analysis of the mean value was conducted using the factoextra package (Kassambara and Mundt, 2017). Symmetric distribution was tested by the Kolmogorov–Smirnov test using a two-sample package (Dowd, 2022). For the hybrid statistical analysis, seeds from the same flower were considered to belong to the same group. The grouping of samples according to this selection method supported our choice for seed shape analysis (**Supplementary Figure 3**). For the seed cell outline analysis, several cells came from the same seed, and we tested the effect of “Seed” as a random effect and built a mixed model. We performed on the model a nested MANOVA (**Supplementary Table 1**).

### BLAST analysis

Ten representative genes known to control seed size and shape in Arabidopsis and rice were uploaded into Geneious Prime 2023.2.1, from the NCBI nucleotide database (Sayers et al., 2022). The X-chromosome sequence was downloaded and used for local BLAST (Yue et al., 2023). The loci in *A. thaliana* and the genes found in the X chromosome of *S. latifolia* with low E- values are listed in **Table 2**. E-value stands for “Expect value,” and represents the number of hits expected purely by chance; the lower the E-value, the more likely that the hit is real.

**Table 2:** *A. thaliana* loci for genes involved in seed shape identified in the X chromosome of *S. latifolia* through BLAST.

Gene	*A. thaliana* locus	X Chromosome *S. latifolia*	E Value	Hit start	Hit end
***AP2***	AT4G36920	GWHCBIJ00000012	8.52 × 10^−71^	3337982743	337982593
***ARF2***	AT5G62000	GWHCBIJ00000012	2.29 × 10^−09^	366876778	366876674
***SHP1***	AT3G58780	GWHCBIJ00000012	8.15 × 10^−28^	131174848	131175017
***SHP2***	AT2G42830	GWHCBIJ00000012	1.58 × 10^−11^	250746825	250746953
***TTG2***	AT2G37260	GWHCBIJ00000012	1.03 × 10^−07^	104238805	104238971

Hit start/end means the position in the X chromosome where the similarity region is located.

## Results

### Seed morphological changes related with variations in the genome of *S. latifolia*

The results of the statistical analysis of the general morphological descriptors are presented in **Supplementary Table 1** and **Supplementary Figure 4**, including the lateral and dorsal seed views, and seed coat cells. The dorsal view displayed higher heterogeneity, including differences for both classes of GMD, associated with size and shape, and the results related to the dorsal view are summarized in **Figure 1**. The seeds from *S. latifolia* 4× (~♂) in the dorsal view presented the highest values for all GMD related to size, particularly length. This agrees with the higher aspect ratio and lower roundness of the tetraploids. On contrast, tetraploids had the highest circularity values. Additionally, females showed lower solidity than tetraploids. No statistical differences were found between the male and female seeds.

The dorsal outlines were subjected to additional symmetry tests. The extraction of the EFD allows symmetry analysis. The mean symmetry index for all the seeds was over 0.9, and this index ranged from 0 (asymmetric) to 1 (symmetric), which indicates a low variation. The symmetric distribution of the symmetry index in *S. latifolia* 2× (♀ + ♂) did not follow the same pattern as the other genotypes (**Figure 1B**). The tetraploid and male seeds showed directional asymmetry to the right and left, respectively. An additional experiment for sex identification in a population of 50 *S. latifolia* 4× (~♂) seeds provided only one female seed, which means 98% of male seeds in the *S. latifolia* 4x (~♂) seed analysis. Female seeds, on the other hand, show a different type of asymmetry, known as antisymmetry. Statistical differences were found between male and female distributions; additionally, males showed differences from the rest.

### Identification of genes involved in seed size located in the X chromosome

The possibility of detecting genes involved in seed shape in the recently published X chromosome of *S. latifolia* was tested. Ten relevant genes were identified in *A. thaliana* and rice and aligned using BLAST: *SHP1 (SHATTERPROOF1)* and *SHP2 (SHATTERPROOF2)*, which are involved in regulating valve margin development in the seed pods. *INDEHISCENT (IND)* is responsible for the formation of seed coat and pod dehiscence. *AUGMIN SUBUNIT4 (AUG4)* is associated with seed elongation and shape control in *Arabidopsis*. *IKU1 (INNER NO OUTER1)* affects seed shape and embryo development in Arabidopsis. *TTG2 (TRANSPARENT TESTA GLABRA2)* regulates seed coat development. *AUXIN RESPONSE FACTOR2 (ARF2)* is involved in hormone signaling pathways that affect seed development and shape. *APETALA2 (AP2)*: *AP2* is a transcription factor that plays a role in controlling seed size and shape. *DA1 (DAMAGED DNA BINDING PROTEIN1)* is a regulator of cell expansion and influences seed size and shape. *GW5 (GRAIN WIDTH5)*: identified in rice, the *GW5* gene has homologs in Arabidopsis that are involved in seed size determination. From the 10 genes analyzed, we confirmed five genes with a potential effect on seed size and shape located on the X chromosome of *S. latifolia* (**Table 2**).

### Seed morphology in *S. latifolia* hybrids

This section includes the results of the analysis of *S. latifolia* hybrid seed shape and cell morphology. The analysis was based on multivariate analysis (PCA). Seeds from *S. latifolia* 4× (~♂) were included as outgroups due to the similarities shown in the previous analysis.

#### Seed shape in *S. latifolia* hybrids

Three different morphometric approaches were used to evaluate seed shape in different hybrid combinations, and multivariate analysis was based on GMD, *J* index values, and EFA (**Figure 2**). Statistical analysis revealed genotype-dependent differences for all datasets used in each PCA (**Supplementary Table 1**).

In the first principal component analysis, PC1 showed all the hybrids in the negative axis except the hybrid Sd F × Sl M 1, which is in agreement with the smaller size of the seeds resulting from most of the crosses. *S. latifolia* 2× and 4× (~♂), and *S. dioica* were concentrated on the positive axis (**Figure 2B**). There was a common distribution in the positive axis in PC2 for *S. dioica* and the hybrid genotypes, where *S. dioica* was the maternal genotype. On the other hand, *S. latifolia* 2× and 4× (~♂), as well as hybrids where *S. latifolia* was the maternal genotype, were found on the negative axis. PC1 suggests differences between single-species genotypes and hybrids, while PC2 indicates the importance of the maternal genome in the hybrids for these two species of *Silene*. According to the variables, roundness, both for lateral and dorsal views, was the measurement with more influence in the hybrids where *S. dioica* was the maternal genome. The aspect ratio, also for both views, was the parameter with the greatest influence in the hybrids where *S. latifolia* was the maternal genome. The statistical analysis based on the GMD supported the PCA distribution and showed differences in aspect ratio; higher for those genotypes where *S. latifolia* was the maternal genome, and roundness was higher for those genotypes where *S. dioica* was the maternal genome (**Supplementary Figure 4**; **Supplementary Table 1**). Multivariate analysis based on the *J* index (similarity to geometric models) for both lateral and dorsal views, showed a similar distribution to GMD (**Figure 2C**), and the statistical analysis showed similarity with the maternal seed. In addition, hybrids, mainly those generated using *S. dioica* as the maternal genome, were closer in the same quadrant. This suggests that the *J* index offers more sensitive information to evaluate not only the parental sex in the crosses but also to analyze different degrees of hybridization between these species based on their similarity to different geometric models.

The PCA based on EFA performed with the data obtained from lateral and dorsal seed outlines (**Figure 2D**) yielded results similar to those obtained with the GMD and *J* index. However, the distribution in PC1 represents the single-species genomes closer to the origin and the hybrids arranged along both the positive and negative axes. This gradient may reflect the impact of the maternal genome on seed shape. A wide range of different symmetry distributions was observed in the hybrids, but no clear tendency was exhibited, and no parent could be associated with the crosses (**Supplementary Figure 5**).

#### Cell morphology in *S. latifolia* hybrids

**Figure 3:**
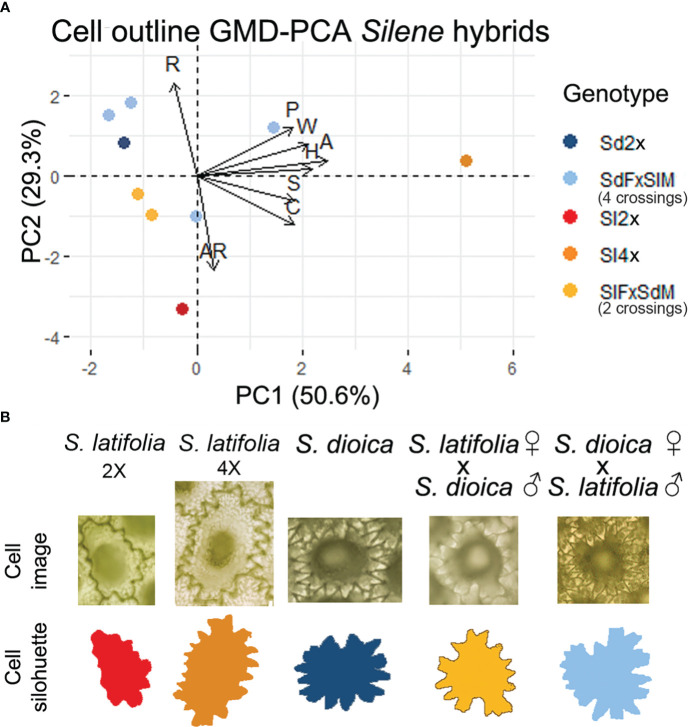
**(A)** Principal component analysis from dorsal cell outline general morphological descriptors **(B)** Representative example seed coat cell image and silhouette from the seed dorsal view for hybrids, parents and *S. latifolia* 4× (~♂). Centroid color code: Red-orange applies to *S. latifolia* and hybrids where *S. latifolia* is the maternal genome. Dark-light blue applies to *S. dioica* and hybrids where *S. dioica* is the maternal genome. Name code: Sl, *S. latifolia*; Sd, *S. dioica*; F, female; M, male.

The GMD of the seed coat cell outlines for each genotype was used to test the variation in the cell morphology of the hybrids. The analysis of cell outlines revealed differences between tetraploids and other genotypes in area, perimeter, length, and width (**Figure 3**; **Supplementary Table 1**; **Supplementary Figure 2**). Significant differences in circularity and solidity were found between the highest values formed by the diploid and tetraploid cells of *S. latifolia* and the other genotypes, particularly seeds resulting from crosses (**Figure 3B**; **Supplementary Figure 4**; **Supplementary Table 1**). The GMD-PCA indicates a similar distribution to that of the seed shape (Compare **Figure 3A** with **Figure 2B**), but size characters are the variables with more influence in PC1, and this can be observed in the contribution plot for each of the principal components (**Supplementary Figure 6**). The contributions of solidity and circularity to PC2 were supported by statistical analysis. Solidity and circularity showed the highest differences, and an interpretation based on this principal component was more informative for shape. Differences in circularity and solidity were observed between crosses with different genotypes. Although seeds derived from crosses of *S. latifolia* males and *S. dioica* females had higher values of these measurements than reciprocal crosses, there was no clear tendency. In addition, the mixed model analysis showed statistical differences from the random effect “Seed,” indicating that it affected the analysis. To remove random effects, one cell from the same dorsal region should be sampled for each seed in future analyses (**Supplementary Figure 4**; **Supplementary Table 1**).

## Discussion

Seeds are reproductive, dispersal, and resistance structures characteristic of angiosperms (Baskin and Baskin, 1998). They are produced inside maternal tissues of sporophytic origin by the double fertilization of the haploid egg and binucleate central cell of the female gametophyte by two sperm cells, giving the embryo and endosperm, respectively. Thus, seeds have tissues from three different origins and genomic compositions: the diploid zygotic embryo and the triploid endosperm of the fertilized seed product, both formed inside the seed coat generated from one or both integuments of the ovule (Spillane et al., 2002). In *Silene*, there is a perisperm that is also of maternal origin, and thus the maternal genotype is predominant (Mohana Rao et al., 1988).

Maternal effects influence various aspects of seed development, including the seed size and shape. From a genetic perspective, in plants with sex chromosomes, the maternal parent contributes genetic material to the seed, including cytoplasmic factors and genes located on the sex chromosomes (Taylor, 1994). We have confirmed five genes with a potential effect on seed size and shape located on the X chromosome of *S. latifolia* (**Table 1**); however, considering the large number of genes involved, many more may be located as well (Li et al., 2019). If a gene on the maternal sex chromosome influences seed coat development or nutrient allocation, it can also affect the size and shape of seeds produced by the mother plant (Ehlers et al., 2016). Moreover, future public availability of the Y chromosome in *S. latifolia* will allow for larger gene identification and comparison between X and Y alleles, given the signs of degeneration detected in most of the Y-linked alleles (Marais et al., 2008; Akagi et al., 2023; Moraga et al., 2023). These facts support the morphological differences found between *S. latifolia* female and male seeds, and agree with previous differences found in *S. latifolia* rosettes in the vegetative state (Zluvova et al., 2010).

All life forms are more or less symmetrical and asymmetry is a consequence of biological processes. Nevertheless, diverse types and degrees of asymmetry can be described and quantified. Some of them are inheritable, such as the directional asymmetry observed in male and tetraploid seeds, which is similar to directional asymmetry in the fore and hind limbs of rabbit fetuses (Breno et al., 2013). On the other hand, in cases of antisymmetry, such as that observed in female seeds, the direction of asymmetry is not inherited. The expression of specific traits might break the pattern because developmental processes have a random factor, and Graham et al. (2010) suggest that the ways in which these symmetries are broken might reflect important evolutionary changes that, in the cases described here, concern *S. latifolia* seed evolution and development. Silene 2× and 4× plants consistently produce 2× and 4× offspring. Previous work on *S. dioica* polyploids indicated that over 90% of males were from tetraploid crosses (Warmke and Blakeslee, 1939), and the sex analysis of *S. latifolia* 4× (~♂) in this study confirmed that this percentage might be over 95%. This may explain the directional asymmetry in tetraploids, similar to males, despite the small number of 4× females included in the population. In addition, the X/Y chromosome ratio can affect the tetraploid seed symmetry. However, our results show differential symmetry distribution, which may involve the development of both sexes through evolutionary changes undergone by both X and Y chromosomes (Hobza et al., 2007; Charlesworth, 2013). Additionally, asymmetry has been proposed as a factor in sexual selection in animals (Møller and Pomiankowski, 1993).

Seeds are complex structures whose sizes and shapes are subject to multiple coordinated regulatory processes. Polyploidy has been shown to have an effect not only on increased seed size (Eliášová and Münzbergová, 2014), but also on many ecophysiological parameters influencing plant environmental adaptation (Stevens et al., 2020). Transposable elements and polyploidy are common mechanisms for increasing nuclear DNA levels across species and seed masses. Nevertheless, TEs are more closely correlated with divergences in genome size than with divergences in other morphological and ecological variables (Beaulieu et al., 2007). Our results confirmed the changes in seed size in the polyploids. These are mainly influenced by length (increased aspect ratio) and are observed in both the lateral and dorsal views. According to the ultrastructure of *Silene* seeds, this may be associated with different embryo/endosperm ratios between *S. latifolia* 2× (♀ + ♂) and 4× (~♂), an aspect that requires further experimentation. We also described an increased size in the cell surface of the polyploid cell, indicating that larger cells, but no increase in cell number, were responsible. In contrast, cell lobes remained unchanged according to solidity, indicating strong seed surface conservation. Together with the morphological analysis by comparison with geometric models, the results show that an increase in size is not accompanied by notable changes in shape, and the statistical analysis indicates that the control of shape is regulated by more restrictive mechanisms than size control, which might be influenced more by environmental factors (Thompson, 1981). Moreover, in *S. latifolia*, the number of flowers depends on leaf area, indicating additional regulation based on the traits of different organs (Steven et al., 2020). The differences in symmetry found in tetraploid seeds indicate the effects of changes in the genome, and these differences have already been identified and used in the analysis of inbreeding and hybridization (Klingenberg, 2008).

*S. dioica* and *S. latifolia* are related species with similar DNA content and chromosome number. In nature, hybrids have often been described and analyzed from genetic and ecological perspectives (Minder et al., 2007; Baena-Diaz et al., 2019; Karrenberg et al., 2019). Nevertheless, the description of the phenotype of artificial hybrids obtained in artificial crosses in the laboratory dates to the decades of fifties and sixties of the past century (Baker, 1950; Van Nigtevecht, 1966), and concentrates on the characteristics that may be of diagnostic use in the recognition of hybrids. In general, the conclusions of these works describe a tendency to matrocliny, i.e., the major relevance of maternal characteristics in the offspring. Baker attributed this to cytoplasmic rather than sex-linked inheritance, and studies of chloroplast DNA variation in *S. dioica* and *S. latifolia* across Europe suggest a history of hybridization and introgression between the two species over broad geographic areas (Baker, 1950; Goulson, 2009). In these reports, a reduced number of seed characteristics were observed, which are of limited utility in the differentiation of hybrids. The measurements related to seed size were similar in both genotypes, *S. dioica* and *S. latifolia*; thus, *a priori* there were no reasons to find differences in the progeny of their crosses. Nevertheless, the seeds resulting from crosses performed with *S. latifolia* females and *S. dioica* males were of reduced size compared to their parents, as well as to the results of reciprocal crosses. Reduced weight in crosses has been described by Baker (1950). In the crosses described here, roundness and aspect ratio were characteristics associated with the maternal genome. Higher values of roundness were associated with *S. dioica* mothers and lower values with *S. latifolia* mothers. Similarly, the same is valid for the aspect ratio in the opposite pattern. Nevertheless, we emphasize that this fact applies only to the lateral view. These two measurements are involved in shape rather than size and are inversely correlated, and the differences between these two measurements could be used to differentiate between *S. latifolia* and *S. dioica* hybrids. In accordance with the results for male and female seeds, seed shape from hybrids can be used to distinguish between parental genotypes, indicating that the maternal genotype is responsible for the seed shape.

Cell shape is clearly influenced by general morphological parameters related to seed size and shape, with solidity and circularity being the main shape descriptors. Defining solidity as the extent to which a shape is convex or concave and circularity as the degree of similarity to a perfect circle (Nowak et al., 2021), based on the cell shape under analysis, we consider solidity to be a better descriptor. However, we conclude that cell shape cannot be associated with any sex. Cell outline analysis of hybrids has been used previously in a *S. acutifolia* × *S. foetida* natural hybrid population in Portugal (Ladero et al., 1999). The authors described cell shape as an intermediate between both parents; however, the description was based on qualitative observation of the cell outline, and no similarity to any of the progenitors was mentioned. Although F1 hybrids usually display a mismatch of characters from their parents, they may not show intermediate morphological characters (Thompson et al., 2021). Flowers in *S. latifolia* (white) and *S. dioica* (purple) hybrids can display a range of color variations, and have been used in the analysis of inherited genetic traits from both parent species (Minder et al., 2007; Karrenberg and Favre, 2008). This morphological characteristic has been used to test the additive genetic inheritance of alleles from both species (Page et al., 2014; Liu and Karrenberg, 2018). Seed morphology offers the possibility of adding a new quantifying tool based on phenotypic characteristics in the analysis of *Silene* hybrids. The asymmetry observed in the hybrids might be explained by the fact that it occurs in species with sex chromosomes, where heterogametic sex often suffers more negative effects from hybridization than homogametic sex, known as Haldane’s rule (Coyne, 1992), and can influence the higher degree of asymmetry in the hybrids where the maternal genotype was *S. dioica*. Paternal parent-of-origin effects control early zygotic development through cell fate determination after first cell division. In Arabidopsis, paternal effects are responsible for asymmetric cell division and elongation (Ohnishi and Kawashima, 2020). A detailed review of heteroplasmy and paternal transmission of mitochondria on *Silene*, specifically in *S. vulgaris*, can be found in Storchova (2011).

In summary, the results from the analysis of the reciprocal crosses between *S. dioica* and *S. latifolia* show a much more complex panorama than just the idea that seed characteristics are the product of the maternal or paternal genotype alone. Although environmental factors, among others, may affect seed morphology, we consider seed shape to be a robust indicator that can be used as a complementary tool for genetic and phylogenetic analysis in *Silene*.

## Data availability statement

The datasets presented in this study can be found in online repositories. The names of the repository/repositories and accession number(s) can be found below: Raw images used in this work are available in Zenodo DOI 10.5281/zenodo.8366177.

## Author contributions

MH: Data curation, Formal analysis, Investigation, Methodology, Resources, Supervision, Validation, Visualization, Writing – review & editing. JJM-G: Data curation, Formal analysis, Investigation, Methodology, Resources, Supervision, Validation, Visualization, Writing – review & editing. EC: Conceptualization, Data curation, Formal analysis, Investigation, Methodology, Project administration, Resources, Software, Supervision, Validation, Visualization, Writing – original draft, Writing – review & editing. RH: Formal analysis, Funding acquisition, Investigation, Methodology, Supervision, Validation, Writing – review & editing. JLRL: Conceptualization, Data curation, Formal analysis, Investigation, Methodology, Project administration, Resources, Software, Supervision, Validation, Visualization, Writing – original draft, Writing – review & editing.

## Supplementary material

Supplementary Table 1 Output of MANOVA for *S. latifolia* sex subpopulation and 2x (♀+♂) Vs 4x (~♂) population and for **Figures 1A**, **2B–D**. ANOVA and Tukey tests for **Figures 1A**, **2B** Nested MANOVA for **Figure 3A**.

## Glossary of morphological terms

**Table:**

Area (A), Measurement of the plane surface of a figure. Perimeter (P), Distance along the continuous line forming the boundary of a closed geometrical figure Length (L), distance along the major axis Width (W), Distance along minor axis Aspect Ratio (AR), L/W Circularity (C), L/W Solidity (S), Relationship between the area of an object to the area of its convex hull Roundness (R), $4A/\pi L^{2}$ *J* index, Relationship between the area shared between an object and a geometric model to the area total (shared and unshared) of both figures. Code used in multivariate analysis: For the seed lateral view, L suffix was added to each abbreviation; Area lateral view, AL. The same code was used for the seed dorsal view adding D suffix to each abbreviation. For the cell outlines the abbreviations of the terms were used.
