# Supplementary material for: Seed shape and size of Silene latifolia, differences between sexes, and influence of the parental genome in hybrids with Silene dioica
Source: Front Plant Sci. 2024 Mar 11;15:1297676. doi: 10.3389/fpls.2024.1297676 (PMC10961389; doi:10.3389/fpls.2024.1297676)
Supplement: Supplementary file 2 [file DataSheet_1.pdf]

Supplementary Material

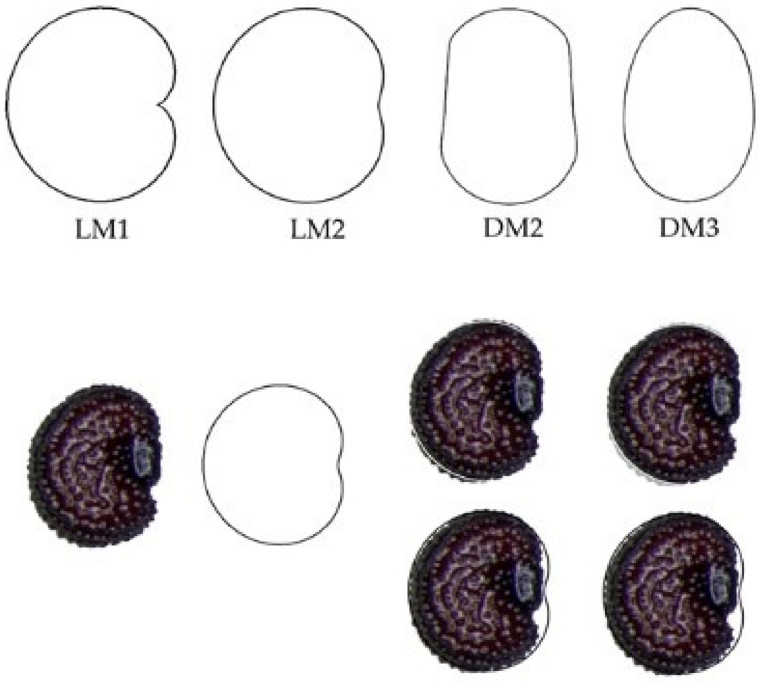

Figure S1. Top: Lateral cardioid models and dorsal superellipse models used for seed shape quantification. Bottom: An example of *J* index quantification by comparison with geometric models. From left to right: Seed image, geometric model (LM2), seed image with model superimposed in white (above) and black (below), shared (above) and total area (below).

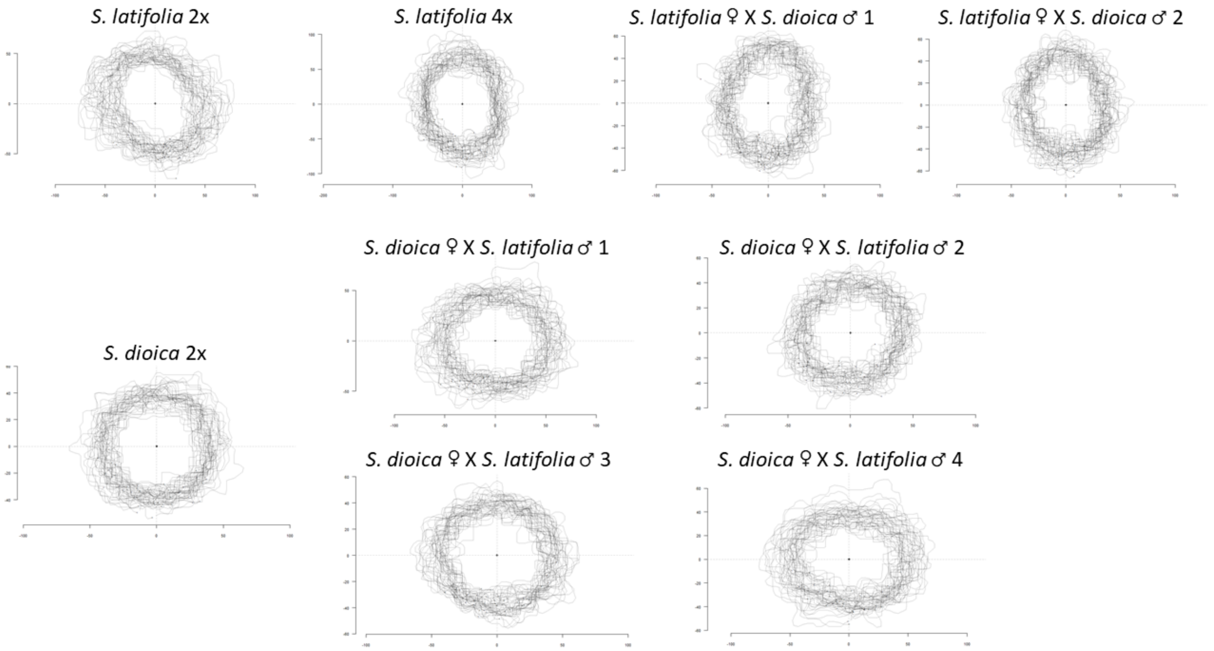

Figure S2. Stacked cell coat outlines used for each genotype in the multivariate analysis. After centering and scaling a similar morphology can be observed.

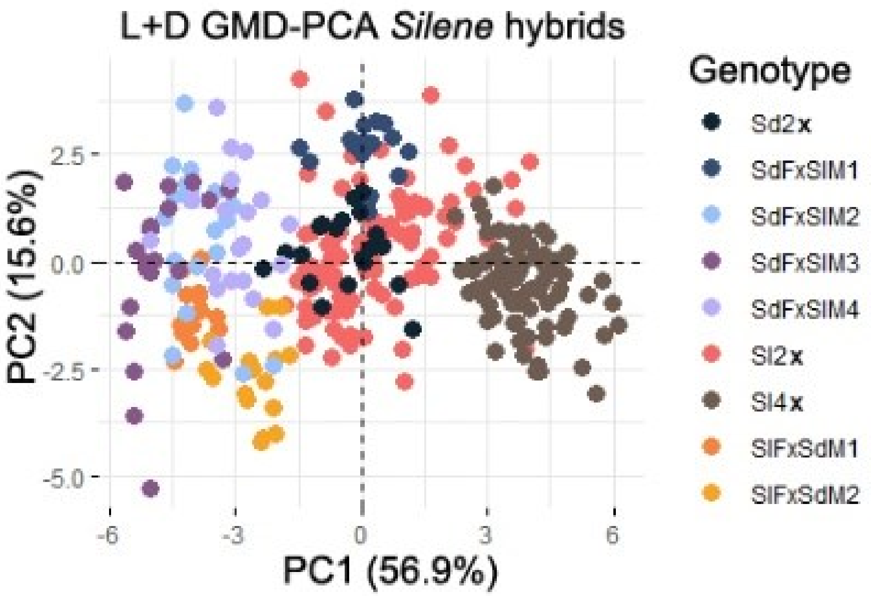

Figure S3. Principal component analysis from lateral plus dorsal view for general morphological descriptors for *Silene* hybrids and parents. Each genotype is associated to a color to show how the genotypes group. *S. dioica* diploid seeds and hybrid seeds originated with *S. dioica* as maternal genotype share the range from dark blue to purple color. *S. latifolia* diploid seeds and hybrid seeds originated with *S. latifolia* as maternal genotype share the range from red, brown to orange color. Name code: Sl = *S. latifolia*, Sd = *S. dioica*, F = female, M = male.

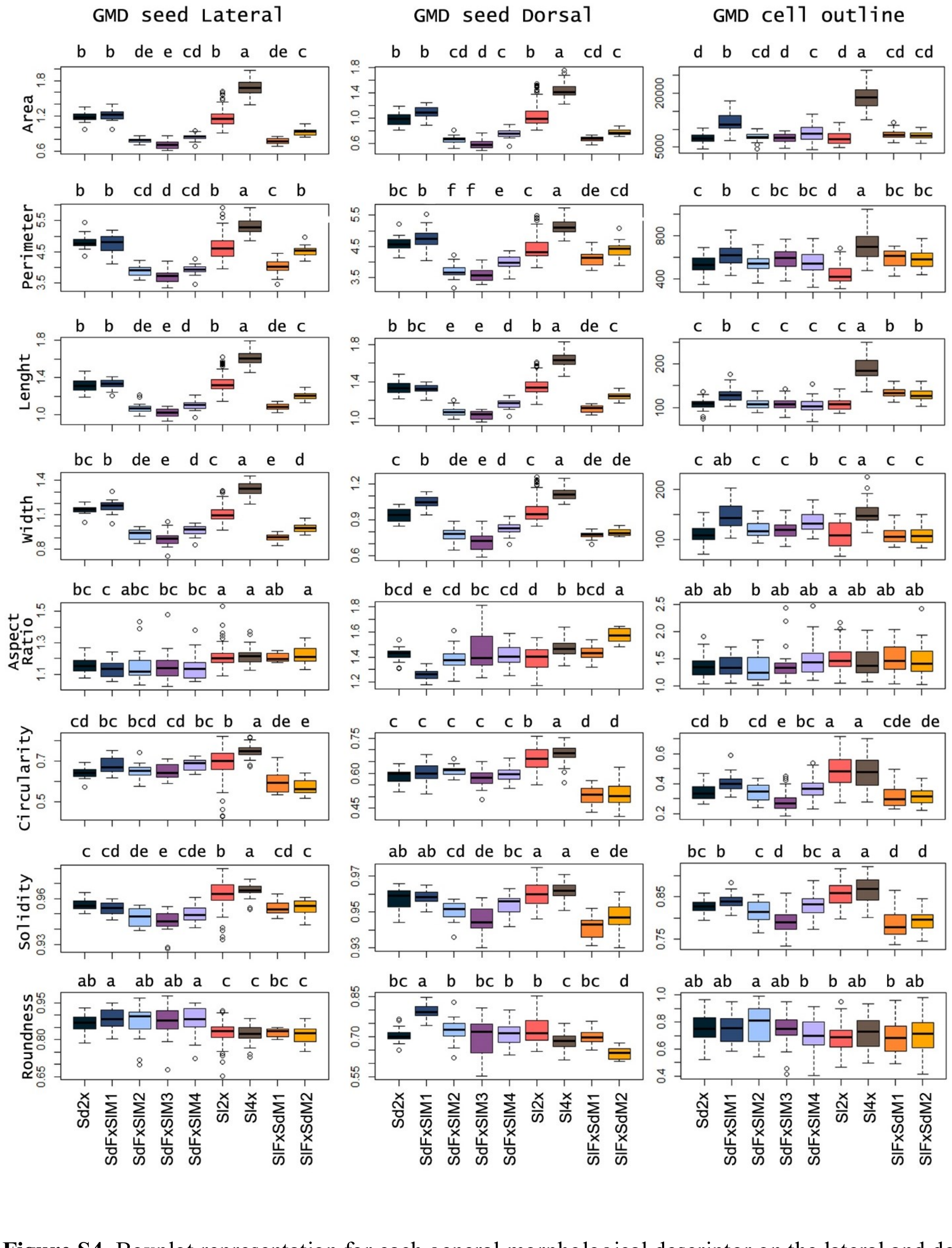

Figure S4. Boxplot representation for each general morphological descriptor on the lateral and dorsal view of *S. latifolia* seeds. Different letters in each individual plot mean statistical differences according to ANOVA and Tuckey tests, p-value <0.05.

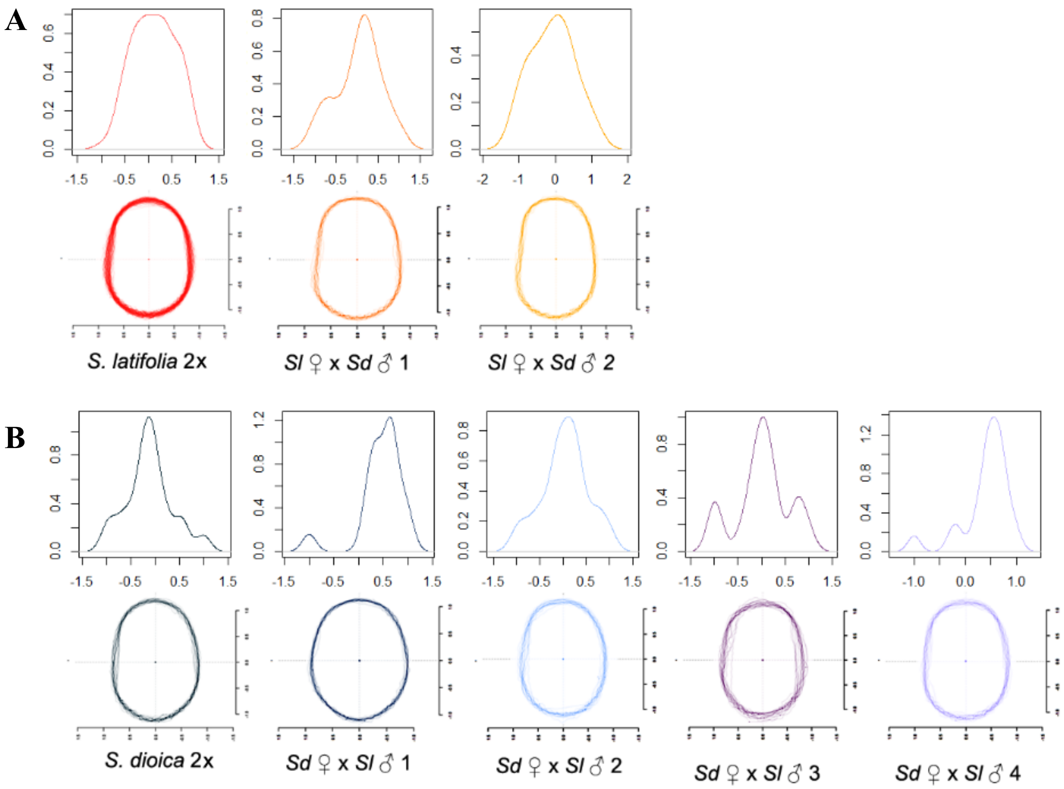

Figure S5. Density plot (up) from the symmetry data and scaled and centered outline stack (bottom) for the dorsal view of **(A)** diploid *S. latifolia* seeds, and hybrid seeds originated with *S. latifolia* as maternal genotype. **(B)** *S. dioica* diploid seeds and hybrid seeds originated with *S. dioica* as maternal genotype. Code: Sl = *S. latifolia*, Sd = *S. dioica*

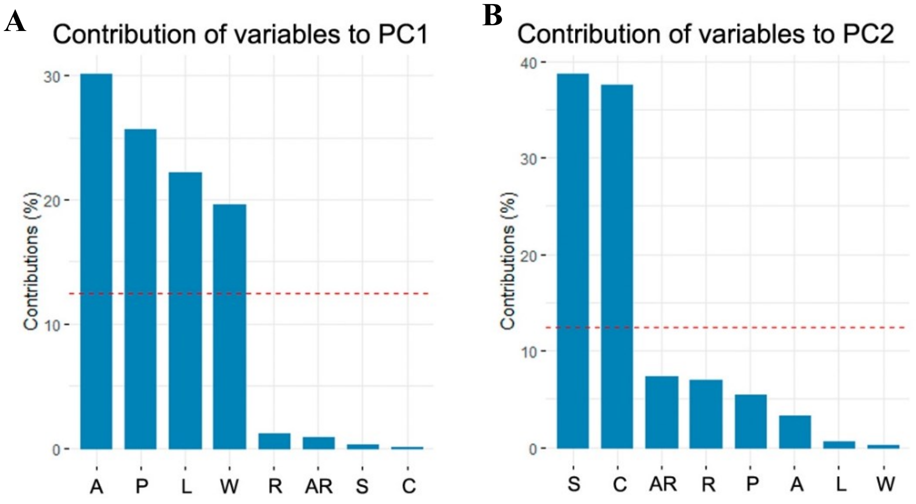

Figure S6. Variable contributions in the principal component analysis from dorsal cell outline general morphological descriptors. **(A)** Contributions to PC1. **(B)** Contributions to PC2.
